# A Rare Case of Juvenile Polyposis Syndrome in a 13-year-old Girl from a Rural Area

**DOI:** 10.7759/cureus.4567

**Published:** 2019-04-30

**Authors:** Murk Lakhani, Zainab Mohsin, Sarmad Pirzada, Imrana Zulfikar

**Affiliations:** 1 Surgery, Dow University of Health Sciences (DUHS), Karachi, PAK; 2 Internal Medicine, Dow University of Health Sciences (DUHS), Karachi, PAK

**Keywords:** juvenile polyps, polyposis, intussusception, colorectal cancer, pan proctocolectomy, ileoanal anastomosis

## Abstract

Juvenile polyposis syndrome (JPS) is a non-cancerous benign growth predominant in a young population with an estimated incidence of one in 1, 00,000 to 1, 60,000 per year. It is a rare genetic presentation, which can occur sporadically as well. There is a 39% evident risk of developing colorectal carcinoma. Herein, we present an unusual case of a 13-year-old girl from a rural area with a negative family history of juvenile polyposis, who had complaints of rectal prolapse and rectal bleeding which were more pronounced after defecation. Her contrast computed tomography (CT) scan revealed a distended large bowel studded with multiple juvenile polyps throughout, the largest of which was detected on the mid rectum. Colo-colic intussusception was also found due to a large polyp at the hepatic flexure, inferiorly extending up to ascending colon and caecum. Pan proctocolectomy with ileoanal J pouch anastomosis was performed, bearing in mind the risk for colorectal cancer and her general state of health.

## Introduction

Juvenile polyposis syndrome (JPS) is characterized as a non-cancerous benign growth, which occurs predominantly in the young population. It commonly presents with anemia, abdominal pain, diarrhea and bleeding per rectum; however, in few case presentations, it may be accompanied by intussusception, intestinal obstruction or polyp prolapse (through the anal sphincter). Numerous juvenile polyps constitute an increased risk of intestinal cancer if they are more than five in number [1].

The colon is the most common site but it can be located anywhere along the gastrointestinal tract. JPS is typically recognized by the age of 30 years, while the average presenting age is 9.5 years [2]. It is a rare condition which can either be familial with an autosomal dominant origin or sporadic; with an estimated incidence of one in 1, 00,000 - 1, 60,000 per year [3]. Whereas, the incidence rate of JPS has not been reported yet in Pakistan. The progressive risk of developing colorectal carcinoma is 39% in patients with JPS, with moderate risk by 34 years of age [4]. The increased risk for the transformation of JPS to neoplastic state is due to its supposed stromal origin as opposed to epithelial [4]. Inevitably, diagnosis for polyposis requires upper and lower gastrointestinal tract endoscopy, polyp excision, and cytology; nevertheless, we present an unusual case of JPS, which originated in a resource-limited area of diagnosis and treatment.

## Case presentation

A 13-year-old girl from a rural area was admitted to the Surgery Department of Civil Hospital, Karachi, Pakistan with a history of painful rectal bleeding and protrusion of a red mass through anus after defecation for the last two years. Initially, the mass was small in size although it progressively increased in size, which protruded on defecation and had to be reduced manually. It was associated with fresh bleeding and pain after passing stool. She developed pallor and generalized body weakness over time. However, the patient had no significant history of nausea, vomiting, diarrhea, constipation, blood transfusion or any surgery. Her family history was also negative for colonic polyps and cancer. She was taken to a local general physician in her area but her condition did not improve. Then, she was referred to tertiary care hospital for further management.

**Figure 1:**
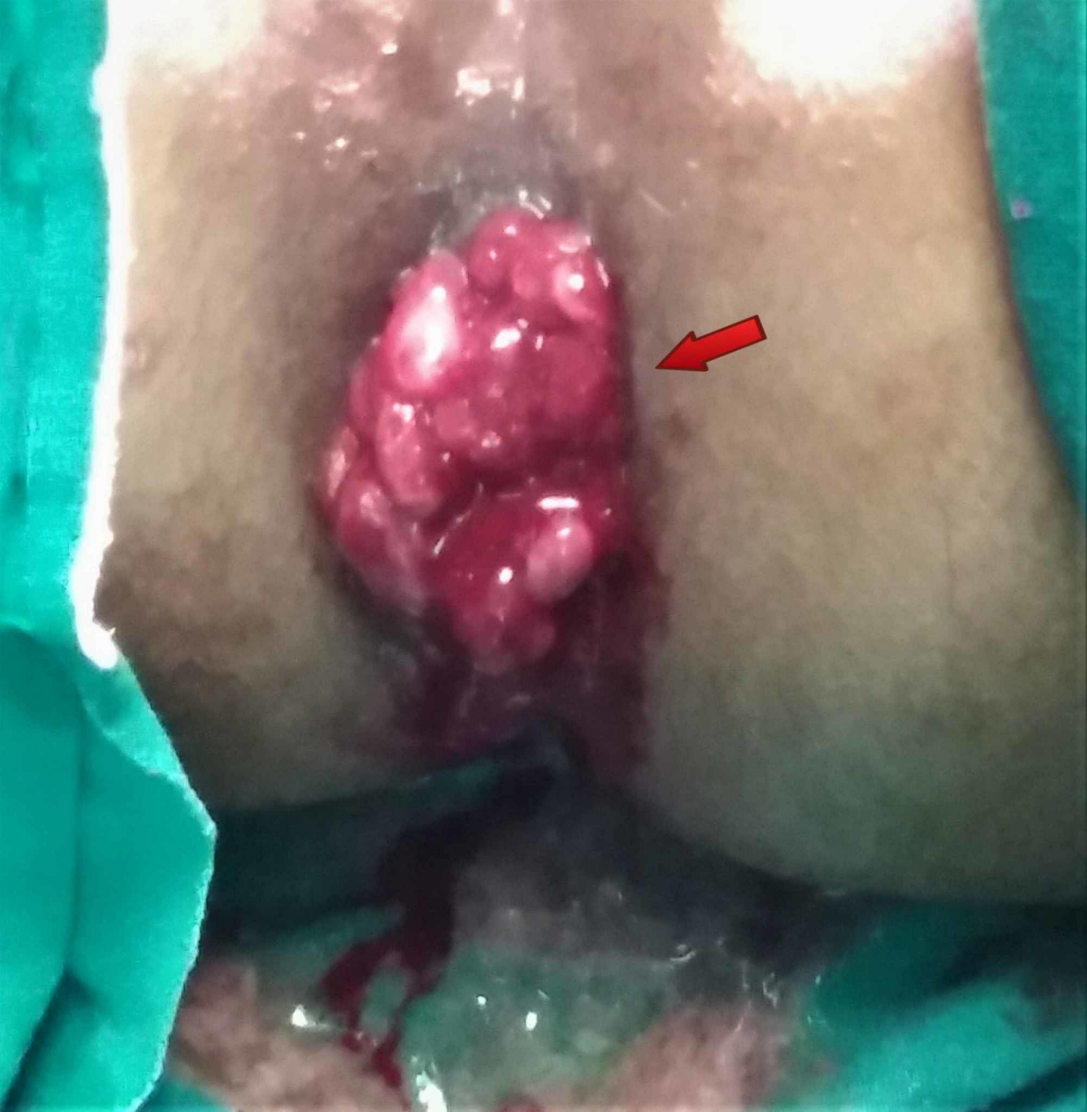
Blood stained polypoid masses prolapsing out of anal canal

On general physical examination, she was ill-looking and lethargic. Her pulse was 90 beats/minute; blood pressure 90/60 mmHg; respiratory rate 20 breaths/minute; afebrile. There were no signs of jaundice, cyanosis, clubbing or koilonychia. On abdominal examination, her abdomen was soft, mildly tender with audible gut sounds. The digital rectal examination was performed which revealed friable, easily prolapsed and bloodstained multiple pedunculated polypoid masses with normal anal sphincter tone and anal canal (Figure 1). All other systemic examinations were unremarkable.

**Figure 2:**
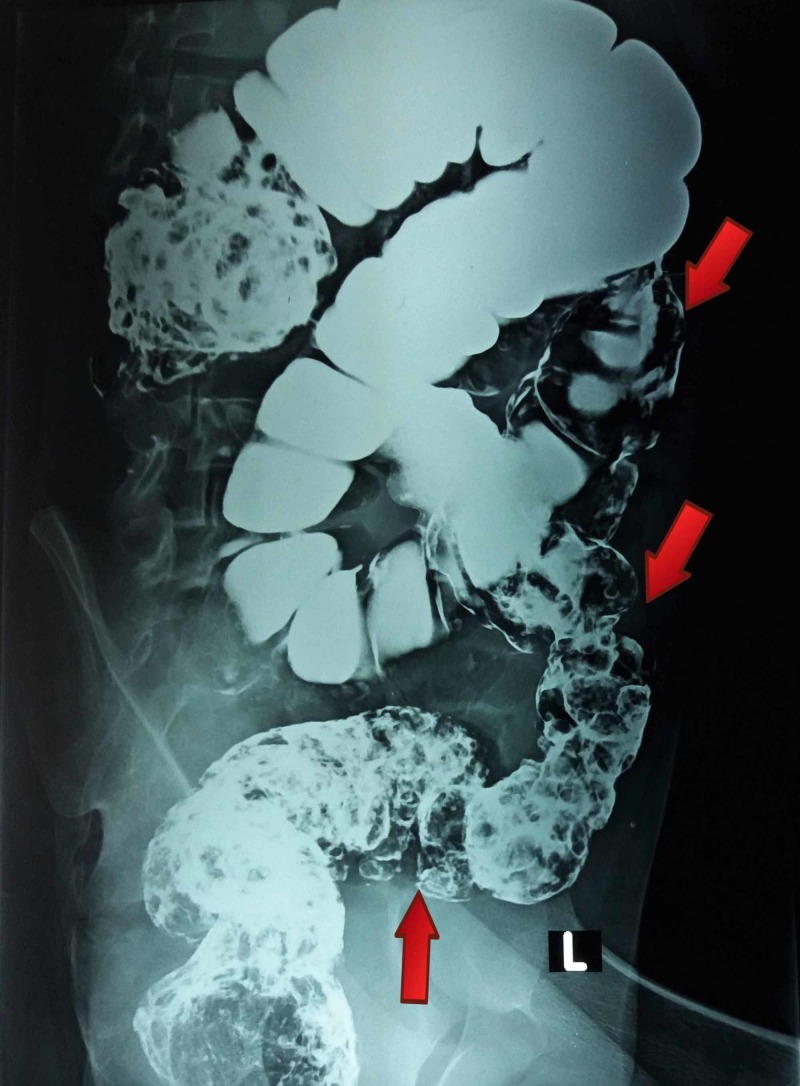
Barium enema showing multiple filling defects dispersed diffusively in the large gut up to rectum

**Figure 3:**
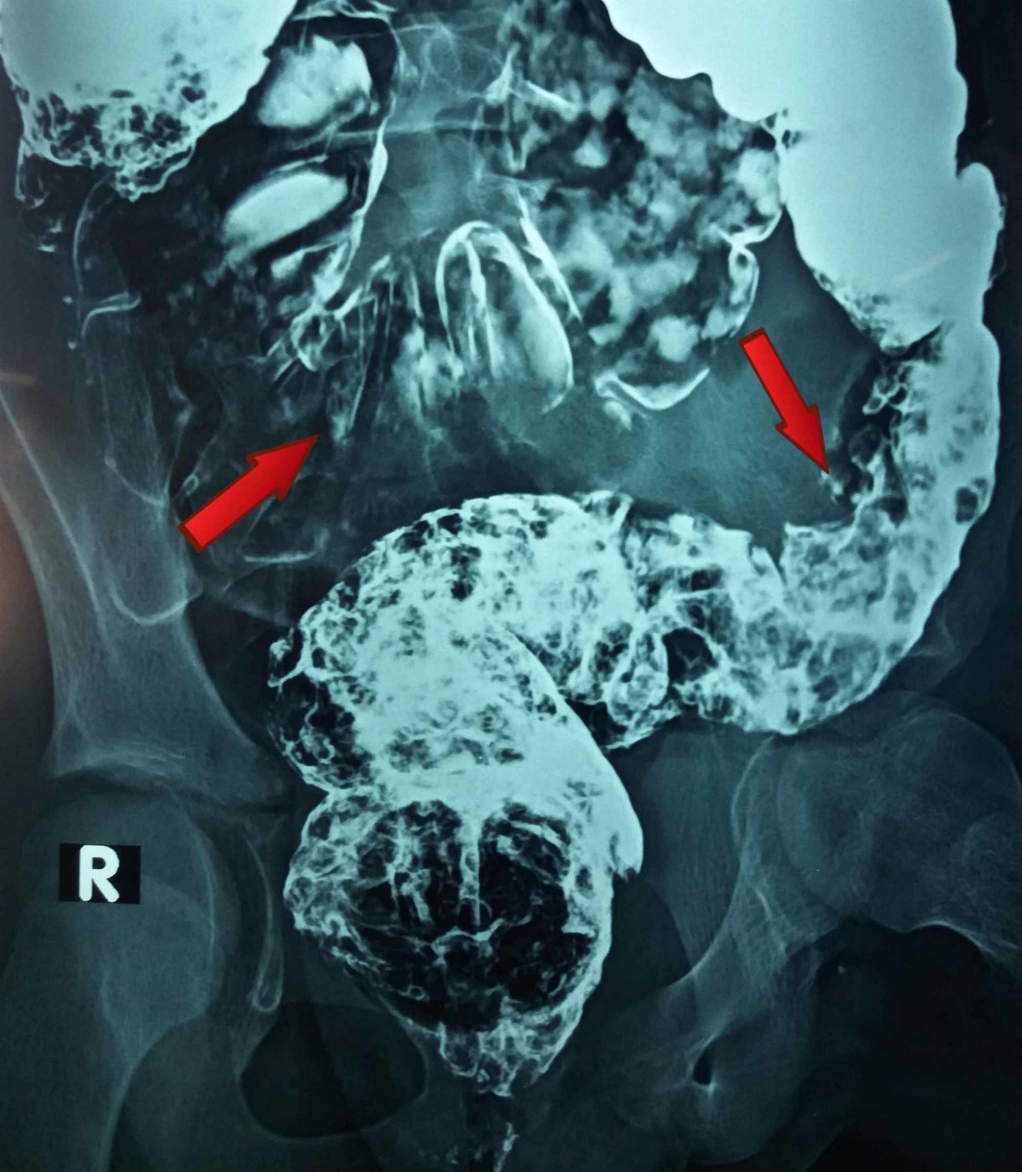
Barium enema showing multiple filling defects dispersed diffusively in the large gut up to rectum

The full blood count showed hypochromic microcytic anemia with hemoglobin of 10 g/dl, packed cell volume of 28.6% and a mean corpuscular volume of 66.8 fl. The white blood cells count indicated neutrophilic leukocytosis of total leukocytes 16.7x109/L and neutrophils 79%. Other laboratory tests were within normal ranges. The chest X-ray and ultrasound scan of the abdomen were normal. Small multiple erosions in fundus were found on upper gastrointestinal tract endoscopy while the rest was unremarkable. Colonoscopy illustrated hundreds of pedunculated polyps throughout the colon. Double contrast barium enema was performed which demonstrated various filling defects, scattered diffusely in the large bowel up to the rectum (Figures 2-3).

Contrast computed tomography (CT) scan of abdomen depicted distended large bowel which was studded with numerous, pedunculated and broad-based soft tissue density filling defects of variable sizes throughout the transverse colon, descending colon, sigmoid colon, and rectum; largest of them found in the mid rectum was measured 2.3x2.07cm. A large, irregular, heterogeneously enhancing polypoidal like mass lesion was seen at the hepatic flexure, inferiorly extending up to the ascending colon and caecum, measuring up to 3.9x4.9x3.0 cm (CCxTVxAP dimensions), also causing colo-colic intussusception. Multiple enlarged solid enhancing lymph nodes were found and the largest of them measured 1.3x1.0 cm at the ileocolic region. Histopathology report confirmed benign juvenile polyposis, showing a number of polyps with cystically dilated, hyperplastic glands, lined by tall columnar epithelium with basally placed nuclei. Some glands were filled with fibrin and intervening stroma was inflamed and showed congested blood vessels. Most of the polyps were covered by granulation tissue but indicated no evidence of dysplasia or malignancy.

**Figure 4:**
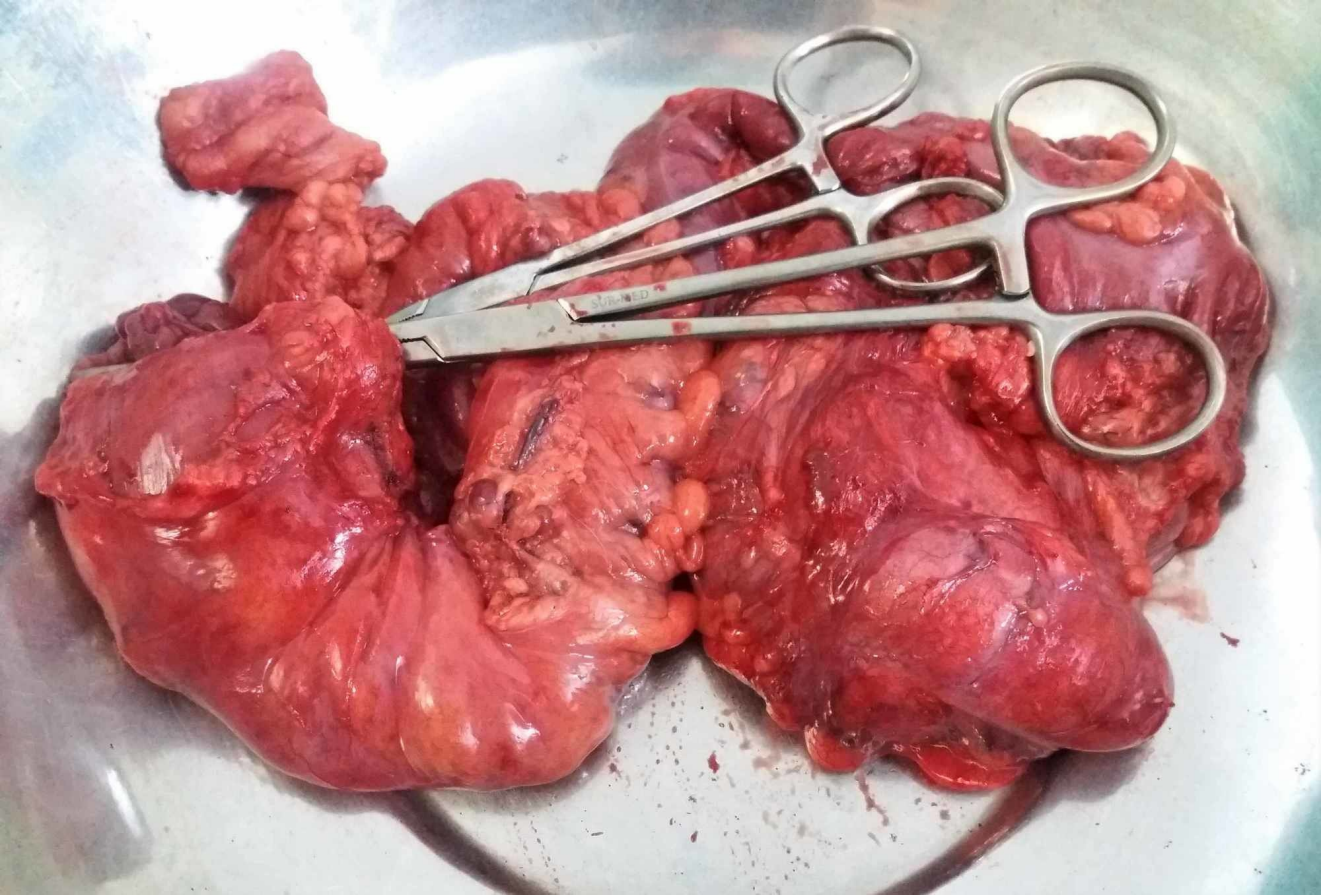
Colectomy specimen revealing multiple polyps throughout the colon and rectum

Genetic testing was unavailable at our hospital. A pan-proctocolectomy with ileoanal J pouch anastomosis was planned. After consent and counseling on long-term risk of colorectal cancer associated with polyposis; exploratory laparotomy was performed and numerous polyps in colon and rectum were found. The entire colon and enlarged lymph nodes were resected (Figure 4) and ileoanal J pouch anastomosis was made. Postoperatively family was counseled on proper fluid intake, soft and small meals, and hygiene, given their rural background. The patient was also informed to have regular checkups and to take part in routine tasks actively. There were no active complains within one-month follow-up post discharge. 

## Discussion

JPS is one of the hamartomatous polyposis syndromes usually associated with mutations in either of the two genes namely BMPR1A and SMAD4, linked to the TGF-B/BMP signaling pathway [3]. It may arise sporadically within families or may be inherited as an autosomal disorder. Significantly, “Juvenile” in JPS distinguishes the polyp’s type rather than the age group most commonly affected [5]. Despite the benign polyps, malignancy is always a suspected risk in JPS which increases the chances of gastrointestinal cancer, ranging from 9-50% [5].

JPS is diagnosed when a patient has any one of these findings (Jass criteria): polyps in colon or rectum greater than five in number, juvenile polyps throughout the gastrointestinal tract or any number of juvenile polyps found in one or more family members [5-6]. In this case, the patient fulfilled two of these three findings. Moreover, she presented with severe bleeding after defecation and reducible rectal mass. JPS was diagnosed when multiple polyps were observed in the gastrointestinal tract (caecum to rectum) on colonoscopy and CT scan of the abdomen. It was recognized as one of those cases with new sporadic mutations found in about 25-50% of the patients with JPS, which is more common than familial JPS [7].

In this case, the largest polyp was measured about 2.3x2.07 cm in the mid-rectal region. In similar studies, it was proposed that polyps within the size of 1-2.9 cm have mildly dilated glands and moderately dysplastic epithelium with further sequential changes may progress into an adenoma leading to the formation of adenocarcinoma [8]. On radiography, a large heterogeneously enhancing polypoidal mass like lesion was seen at the hepatic flexure resulting in colo-colic intussusception which is an infrequent occurrence due to the fixed retroperitoneal position of the descending colon and hasn’t been seen in many cases to date [9].

Our patient belonged to the rural side of the country where the likelihood of survival and treatment from any rare or complicated disease is minimal. It is a case originating from an ineffectual and resource-limited setting. Due to these constraints and delayed presentation; the cases subsequently culminate poorly for the patients with low chances of survival or compromised quality of life. A case of a 7-year-old boy reported in India, with progressive rectal prolapse and bleeding for five months and was treated with total proctocolectomy with ileoanal anastomosis. However, due to the presentation in a well-established and efficient environment, the patient was found to be doing well on a two-year follow-up after his surgery [3].

The treatment of choice in the symptomatic patients includes surgical colectomy and ileorectal anastomosis including surveillance endoscopy of the remaining rectum [10]. However, the definitive treatment in our patient was pan proctocolectomy with ileoanal J pouch because of the pre-malignant polyps and hyperplastic dilated glands that were observed in the whole rectal region. 

## Conclusions

Despite digital rectal examination being of great value to assess such cases, the unavailability of important diagnostic tools like colonoscopy, CT scan in rural areas delays the whole process of management. Hence, measures need to be taken in order to provide better chances of survival to this part of the population. With a better educational background, awareness, and resources, polyps and early stage cancer can be identified and removed.
